# Sustained clinical stability with telitacicept-containing maintenance therapy following glucocorticoid and IVIG induction for lupus enteritis with autoimmune hemolytic anemia: a case report

**DOI:** 10.3389/fimmu.2026.1872883

**Published:** 2026-09-09

**Authors:** Jinhui Tan, Hai Huang, Linghua Tan, Jianlong Zhu

**Affiliations:** 1 Department of Rheumatology and Immunology, People’s Hospital of Longhua, Shenzhen, Shenzhen, Guangdong, China; 2 Department of Health Management, People’s Hospital of Longhua, Shenzhen, Shenzhen, Guangdong, China; 3 Department of Health Management, Jiangmen Wuyi Hospital of Chinese Medicine, Jiangmen, Guangdong, China; 4 Department of Pathology, People’s Hospital of Longhua, Shenzhen, Guangdong, China

**Keywords:** anemia, hemolytic, autoimmune, glucocorticoids, lupus erythematosus, systemic, telitacicept, vasculitis

## Abstract

Lupus mesenteric vasculitis (LMV) is a rare but potentially life-threatening complication of systemic lupus erythematosus, with reported mortality rates of up to 13.4% despite aggressive immunosuppression. Standard management relies on high-dose glucocorticoids (GCs) combined with cyclophosphamide (CYC) or mycophenolate mofetil (MMF); however, prolonged glucocorticoid dependence and disease recurrence remain significant clinical challenges. Telitacicept, a recombinant transmembrane activator and calcium-modulating cyclophilin ligand interactor (TACI)-Fc fusion protein that simultaneously neutralizes B-lymphocyte stimulator and a proliferation-inducing ligand, has demonstrated efficacy in active SLE, yet evidence for its use in LMV is limited to a single case report. We describe a 27-year-old female with severe, glucocorticoid-dependent LMV complicated by autoimmune hemolytic anemia (AIHA), who received pulse methylprednisolone (MP) and intravenous immunoglobulin (IVIG) for acute induction, followed by telitacicept 160 mg weekly as part of a combination maintenance regimen. After conventional induction, telitacicept was associated with successful prednisone tapering from 60 mg/day to 10 mg/day over approximately 6 months without disease flare, accompanied by sustained normalization of hemoglobin and complement levels, and radiological improvement. This case represents the second report of telitacicept for LMV and the first to document sustained resolution of AIHA maintained during telitacicept-containing therapy. The induction with pulse MP followed by telitacicept maintenance and accelerated steroid taper” strategy may offer a promising therapeutic option for refractory LMV warranting further prospective investigation.

## Introduction

Systemic lupus erythematosus (SLE) is a prototypical autoimmune disease characterized by aberrant B-cell activation, autoantibody production, and immune complex-mediated multi-organ damage (1). Among its severe complications, lupus mesenteric vasculitis (LMV), also termed lupus enteritis, represents a rare but potentially life-threatening gastrointestinal manifestation, with reported mortality rates reaching 13.4% despite aggressive immunosuppressive therapy (2, 3). Standard management of LMV relies on high-dose glucocorticoids (GCs) combined with either cyclophosphamide (CYC) or mycophenolate mofetil (MMF) (2, 3); however, sustained GCs dependence and disease recurrence remain significant clinical challenges, underscoring the urgent need for effective steroid-sparing strategies (2, 4).

B lymphocytes and their regulatory cytokines, B-lymphocyte stimulator (BLyS, also known as BAFF) and a proliferation-inducing ligand (APRIL), play a central pathogenic role in SLE by driving autoantibody production and perpetuating immune dysregulation (5, 6). Belimumab, a monoclonal antibody that selectively neutralizes BLyS, was the first biologic approved for SLE and established proof-of-concept for B-cell pathway modulation (7, 8). However, a substantial proportion of patients fail to achieve adequate responses, potentially because APRIL-mediated long-lived plasma cell survival pathways remain intact (5). Telitacicept, a recombinant transmembrane activator and calcium-modulating cyclophilin ligand interactor (TACI)-Fc fusion protein, simultaneously neutralizes both BLyS and APRIL, thereby suppressing a broader spectrum of B-lineage cells—from transitional and mature B cells to long-lived plasma cells—offering a theoretical mechanistic advantage over selective BLyS inhibition (6, 8). Phase 2b and phase 3 clinical trials, along with real-world studies, have demonstrated that telitacicept significantly improves disease activity, reduces GCs exposure, and exhibits a manageable safety profile in patients with active SLE (9–11). Furthermore, a recent study specifically evaluating telitacicept in SLE patients with hematological involvement reported significant hemoglobin improvement, suggesting its efficacy in autoimmune hemolytic anemia (AIHA) (12).

Despite these advances, evidence for telitacicept in the treatment of LMV remains extremely limited. LMV was not explicitly represented in the pivotal telitacicept trials (9, 10), and current treatment guidelines do not address the role of dual BLyS/APRIL inhibition in this specific manifestation (13). To date, only a single case report has described the successful use of telitacicept in a patient with concurrent LMV and lupus nephritis (14).

Herein, we report the case of a 27-year-old female with severe, glucocorticoid-dependent LMV complicated by AIHA, who achieved rapid disease control with pulse methylprednisolone (MP) and intravenous immunoglobulin (IVIG), followed by sustained clinical stability during telitacicept-containing maintenance therapy. To our knowledge, this represents the second reported case of telitacicept use specifically for LMV and the first to document concurrent AIHA improvement observed in the context of telitacicept-containing maintenance therapy, although the acute response was induced by conventional therapy.

## Case presentation

A 27-year-old Chinese female presented to the Department of Emergency, People’s Hospital of Longhua, Shenzhen on December 30, 2025, with a 14-hour history of acute-onset colicky lower abdominal pain accompanied by diarrhea and vomiting. The pain was initially partially relieved by antispasmodics and analgesics but recurred intermittently, prompting hospital admission. She reported poor appetite over the preceding days but denied fever, headache, or dysuria. Stools were loose, without melena or hematochezia.

The patient was diagnosed with SLE in 2020, with a documented diagnosis that was reportedly based on hemolytic anemia and a positive antinuclear antibody test. However, the original laboratory reports from that admission—including the specific ANA titer, anti-dsDNA, anti-Sm, complement levels, and DAT results—are no longer available. She had since been on oral prednisone, initially 50 mg once daily and tapered over the years to 10 mg twice daily. Her immunosuppressive regimen included MMF (discontinued due to gastrointestinal intolerance) and cyclosporine (stopped in September 2025 because of inadequate response and replaced by tacrolimus (TAC) 1 mg twice daily). At admission, her home prednisone dose was 10 mg twice daily; because a bacterial infection was suspected in the emergency department, it was reduced to 10 mg once daily upon admission. Her other regular medications were TAC 1 mg twice daily, hydroxychloroquine (HCQ) 200 mg twice daily, polysaccharide-iron complex, and calcium supplements. She denied recent arthralgia, photosensitivity, malar rash, oral ulcers, or Raynaud phenomenon. There was no family history of autoimmune disease.

On examination, she was afebrile and hemodynamically stable. Abdominal examination revealed diffuse lower-quadrant tenderness without guarding or rebound, with normoactive bowel sounds. No skin rashes, oral ulcers, or synovitis were noted.

**Figure 1:**
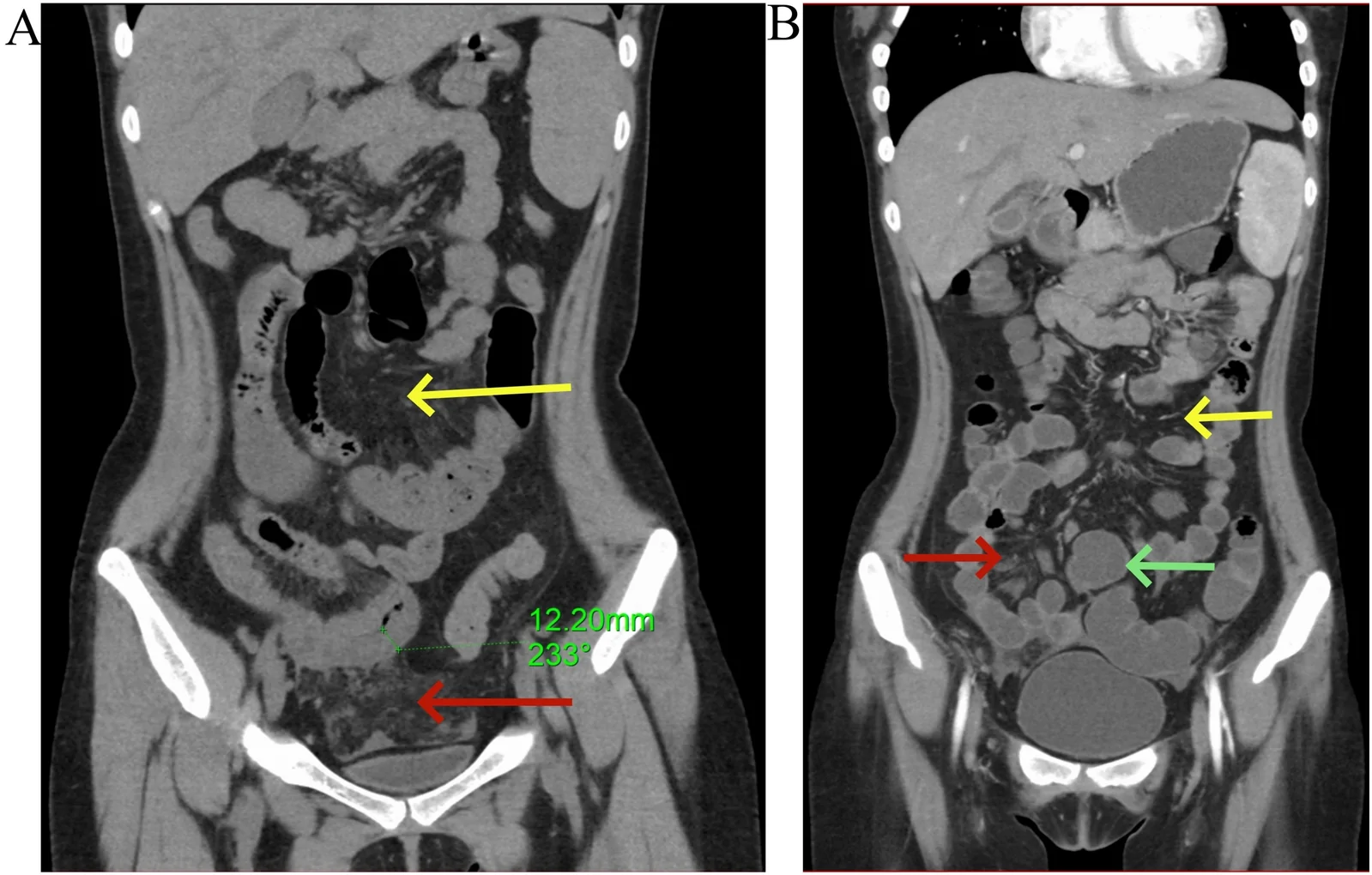
Serial abdominal computed tomography (CT) images. **(A)** Non-contrast CT of the lower abdomen and pelvis obtained on December 30, 2025, at initial presentation, showing engorged mesenteric vessels (yellow arrows), segmental small-bowel wall thickening measuring up to 12.20 mm (green caliper line), and surrounding fat stranding (yellow arrows). **(B)** Contrast-enhanced CT enterography performed on January 10, 2026, after pulse methylprednisolone and intravenous immunoglobulin (IVIG) therapy, demonstrating significant improvement in mesenteric vascular engorgement (yellow arrows), small-bowel wall thickening (green arrows), and perienteric fat stranding (yellow arrows) compared with the baseline scan. The overall findings indicate a favorable radiological response to induction therapy.

Initial laboratory tests showed normocytic anemia (hemoglobin 71 g/L) with a normal white cell count (9.08 × 10^9^/L) and a normal platelet count (331 × 10^9^/L). Hemolysis was indicated by elevated total bilirubin (57.5 μmol/L, predominantly unconjugated), elevated LDH (443 U/L), and a progressive decline in hemoglobin to a nadir of 61 g/L with marked reticulocytosis (absolute reticulocyte count 0.3531×10¹²/L, reticulocyte percentage 23.08%, reticulocyte production index 10.0%, and immature reticulocyte fraction 37.1% [normal <10.5%]), consistent with strong bone marrow compensatory response. Haptoglobin, DAT subtype IgG/C3d, and peripheral blood smear were not available. Urinalysis demonstrated proteinuria (2+), ketonuria (2+), and leukocyturia (1+), without active sediment. Serum potassium was low (3.34 mmol/L). Liver and renal function were otherwise largely unremarkable except for mild hyperuricemia (447 μmol/L). Coagulation, C-reactive protein, and pancreatic enzymes were normal. A plain abdominal X-ray demonstrated short air-fluid levels in the right lower abdomen involving the 5th and 6th groups of small bowel, suggestive of ileus or mesenteric ischemia. Lower abdomen and pelvic non-contrast computed tomography (CT) revealed segmental wall thickening of the distal ileum with surrounding fat stranding, mild mesenteric vascular engorgement (comb-like appearance), and a small amount of pelvic fluid; no target sign (bowel wall enhancement) could be assessed without contrast. Representative baseline and follow-up images are provided in **Figure 1**. Autoantibody screening demonstrated strongly positive antinuclear antibody (>5,000 AU/mL) and positivity for anti-SmD1, anti-U1-snRNP, anti-SSA/Ro60, anti-SSA/Ro52, anti-ribosomal P protein, anti-nucleosome (weak), anti-Ku (weak), and anti-mitochondrial M2 (weak). Anti-dsDNA and anti-phospholipid antibodies were negative. The direct Coombs test (DCT) was strongly positive (4+).

Integrating the acute abdominal presentation, imaging findings, active hemolysis, and serological profile, the consulting rheumatologist diagnosed LMV with AIHA. Other potential causes of enteritis, including infectious enteritis, inflammatory bowel disease, and intestinal obstruction, were considered and excluded based on the clinical context, negative stool cultures, and characteristic imaging findings. The patient was initially managed with bowel rest, intravenous fluids, nasogastric decompression, potassium supplementation, and empirical ceftazidime (1 g q8h). TAC and HCQ were withheld, and prednisone 10 mg daily was continued. She was transferred to the Rheumatology Department on January 2, 2026.

After transfer, repeat laboratory tests on January 2 showed persistent hemolysis: total bilirubin 34.7 μmol/L, direct bilirubin 13.6 μmol/L, indirect bilirubin 21.1 μmol/L, and LDH 334 U/L. Serum albumin was 34.2 g/L. Complement 3 (C3) and C4 were markedly low at 0.40 g/L (reference 0.7–1.4) and 0.05 g/L (0.1–0.4). Two 24-hour urine protein collections quantified 189.05 mg and 396.30 mg, indicating mild proteinuria. Active sediment was absent, and urinary tract obstruction was present; therefore, lupus nephritis was not diagnosed. Urinary tract ultrasonography demonstrated right hydronephrosis and dilatation of the upper right ureter, suggesting distal right ureteral obstruction, possibly due to a stone; the left kidney, left ureter, and bladder were unremarkable. Despite supportive care, hemoglobin declined progressively to a nadir of 61 g/L with reticulocytosis (0.3531×10¹²/L). No packed red blood cell transfusion was administered during the entire course. Baseline screening for hepatitis B, hepatitis C, and tuberculosis was negative.

Given the diagnosis of LMV and uncontrolled hemolysis, pulse therapy with intravenous MP 500 mg daily and intravenous IVIG 20 g daily was administered from January 4 to 8, 2026. Following the pulse, on January 8, bilirubin and LDH decreased, reflecting hemolysis resolution: total bilirubin dropped to 15.3 μmol/L, indirect bilirubin to 8.5 μmol/L, and LDH to 208 U/L. Serum albumin had declined to 27.8 g/L. Hemoglobin rose to 63 g/L on January 8 and further to 108 g/L by January 11. However, by January 15, the pre-discharge hemoglobin was 81 g/L, representing a decrease over the preceding four days. This decline is likely attributable to hemodilution from aggressive intravenous fluid resuscitation and the physiological downward adjustment following the resolution of severe hemolysis, rather than recurrent hemolysis, as bilirubin and LDH remained stable or continued to decline. To assess the mesenteric response, contrast-enhanced CT enterography (CTE) was performed on January 10, demonstrating decreased segmental ileal wall thickening and reduced perienteric fat stranding compared with the emergency non-contrast CT, confirming therapeutic response.

MP was tapered stepwise: 80 mg daily from January 9 to 11, then 40 mg daily from January 12 to 15. Oral TAC (1 mg twice daily) and HCQ (200 mg twice daily) were resumed on January 12. On January 13, telitacicept 160 mg subcutaneous weekly was initiated as a steroid-sparing dual-target (BLyS/APRIL) therapy, and was well tolerated. Abdominal pain and distention resolved completely, and oral intake was gradually advanced.

The patient was discharged on January 15, 2026, on prednisone 60 mg/day, TAC 1 mg twice daily, HCQ 200 mg twice daily, weekly telitacicept 160 mg, and oral potassium chloride. The transition from MP 40 mg/day to prednisone 60 mg/day at discharge was based on the patient’s body weight of 58 kg and an equivalent conversion (1 mg/kg/day of prednisone). Discharge diagnoses were SLE with lupus mesenteric vasculitis, AIHA, hypokalemia, suspected urinary tract infection, and right distal ureteral obstruction.

At outpatient follow-up, she remained clinically well. Potassium supplementation was stopped after January 31 when electrolytes normalized. On February 27, a mild upper respiratory tract infection (URI) prompted temporary cessation of telitacicept and TAC; prednisone was reduced to 50 mg/day and oral levofloxacin given for 7 days, with uneventful recovery. Telitacicept and TAC were resumed on March 14 after confirming clinical stability and a hemoglobin of 117 g/L; complements had normalized (C3 0.85 g/L, C4 0.19 g/L). Prednisone was sequentially tapered: 40 mg/day (March 14), 35 mg/day (March 21), 30 mg/day (March 28), 25 mg/day (April 18), and 20 mg/day (May 1), 15 mg/day (May 10), and 10 mg/day (June 6). By April 18, hemoglobin reached 132 g/L and complements remained normal (C3 0.9 g/L, C4 0.22 g/L), although the direct Coombs test was still positive (4+). Serum albumin had risen to 42.4 g/L. Subsequent liver function and myocardial enzyme tests were unremarkable. No lupus flares, severe infections, or adverse drug reactions occurred throughout follow-up.

On May 24, because urinary protein remained 1+ with leukocyturia (241.3/μL), MMF 0.25 g twice daily was added and subsequently increased to 0.5 g twice daily on June 6; TAC was also increased to 1.5 mg twice daily from June 6. At the last follow-up on July 11, 2026, the patient remained asymptomatic on prednisone 10 mg/day, MMF 0.5 g twice daily, TAC 1.5 mg twice daily, HCQ 200 mg twice daily, and weekly telitacicept 160 mg. Laboratory tests showed hemoglobin 113 g/L, urinary protein negative, C3 0.8 g/L, C4 0.2 g/L, and IgG 10.5 g/L. No disease flares or serious infections were recorded.

**Figure 2:**
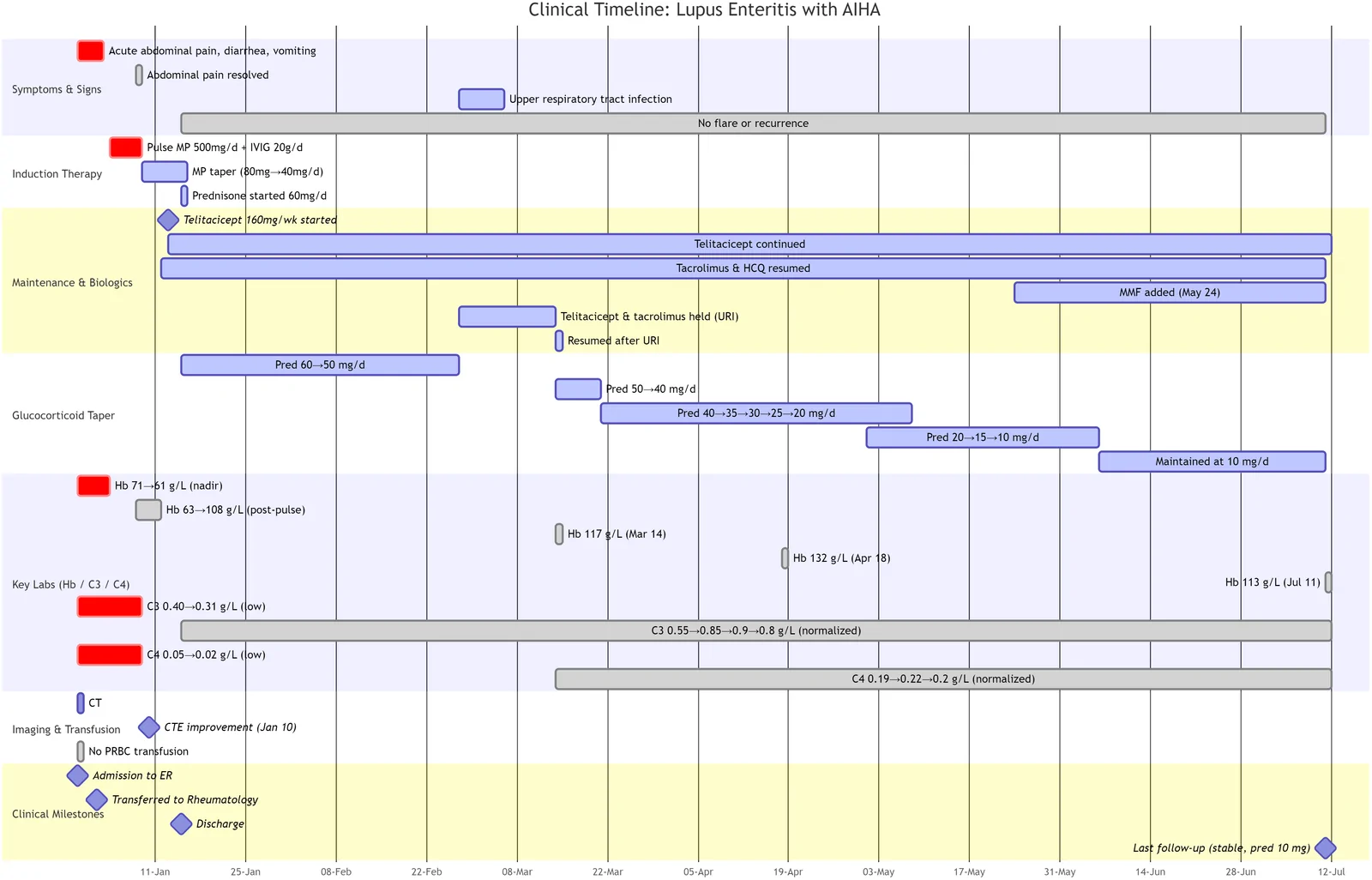
Gantt chart depicting the clinical timeline, therapeutic interventions, laboratory trajectories, and clinical milestones in a patient with lupus mesenteric vasculitis (LMV) complicated by autoimmune hemolytic anemia (AIHA) from admission (December 30, 2025) to the last follow-up (July 11, 2026). The chart illustrates the acute onset of abdominal symptoms, rapid induction with pulse methylprednisolone (MP) and intravenous immunoglobulin (IVIG), early initiation of telitacicept as a steroid-sparing maintenance agent, subsequent glucocorticoid tapering from 60 mg/day to 10 mg/day over approximately 6 months, and the transient interruption of telitacicept/TAC during a mild upper respiratory tract infection. Key laboratory parameters, including hemoglobin (Hb) recovery from nadir 61 g/L to normal levels, complement C3/C4 normalization, and imaging improvement on computed tomography enterography (CTE), are aligned chronologically. No disease flare, transfusion, or severe infection occurred throughout the observation period. AIHA, autoimmune hemolytic anemia; C3, complement 3; C4, complement 4; CT, computed tomography; CTE, computed tomography enterography; ER, emergency room; Hb, hemoglobin; HCQ, hydroxychloroquine; IVIG, intravenous immunoglobulin; LMV, lupus mesenteric vasculitis; MMF, mycophenolate mofetil; MP, methylprednisolone; PRBC, packed red blood cells; TAC, tacrolimus; URI, upper respiratory tract infection.

The rapid glucocorticoid tapering and sustained clinical and hematological stability-–characterized by absence of abdominal symptoms, stable hemoglobin without transfusion, normalized complement levels, and no clinical or available biochemical evidence of recurrent overt hemolysis-–highlight the potential of telitacicept as an effective steroid-sparing option in LMV. Serial IgG, IgA, and IgM levels are shown in **Table 1**; lymphocyte subset analysis was not performed. The longitudinal changes in key laboratory parameters throughout the clinical course are summarized in **Table 1**, and a timeline of the main clinical events is depicted in **Figure 2**.

**Table 1:** Longitudinal changes in key laboratory parameters before and after treatment with pulse methylprednisolone, intravenous immunoglobulin, and telitacicept.

Parameter (Reference range)	2025-12-30 (Admission)	2026-01-08 (Post-Pulse)	2026-01-15 (Pre-Discharge)	2026-03-14 (Follow-up)	2026-04-18 (Follow-up)	2026-07-11 (Follow-up)
Hb (115–150 g/L)	71	63	81	117	132	113
TBIL (0–21μmol/L)	57.5	15.3	17.3	8.9	12.2	10.8
IBIL (0–14 μmol/L)	54.0	8.5	10.5	—	—	—
LDH (120–250 U/L)	443	208	242	—	—	—
C3 (0.7–1.4 g/L)	0.4	0.31	0.55	0.85	0.9	0.8
C4 (0.1–0.4 g/L)	0.05	0.02	0.06	0.19	0.22	0.2
ALB (35–55 g/L)	40.5	27.8	34.3	37.1	42.4	39.9
DCT (Neg)	4+	4+	4+	4+	4+	4+
IgG (8.6–17.4 g/L)	16.2	27.2	21.2	10.3	10	10.5
IgA (1.0–4.2 g/L)	2.6	2	2.4	2.1	1.8	1.6
IgM (0.5–2.8 g/L)	0.9	0.8	1	1	0.8	0.6
dipstick protein (Neg)	2+	1+	—	1+	2+	Neg
WBC (3.5–9.5×10^9^/L)	9.08	4.20	4.70	7.50	7.56	4.77
ALC (1.1–3.2×10^9^/L)	0.27	0.49	0.55	0.83	0.79	0.68

Hb, hemoglobin; TBil, total bilirubin; LDH, lactate dehydrogenase; C3, complement 3; C4, complement 4; ALB, albumin; direct Coombs test, DCT; Neg, negative; WBC, white blood cell count; ALC, absolute lymphocyte count; –, not available.

## Patient perspective

“When the severe abdominal pain started, I was terrified. I knew my lupus was active and I couldn’t endure the pain anymore. Because the pain was so severe, I was also unable to eat, which made me very weak. After the initial treatments (pulse steroids and IVIG) relieved my severe symptoms, my doctors added a new injection, telitacicept, to help reduce my prednisone. I gradually felt better and the weekly injections were easy and I was so relieved the pain never returned. I had a mild cold once and my doctors quickly adjusted my treatment; I felt safe throughout. Now I feel well, my energy is normal, and I’m happy I no longer need high doses of prednisone. I can work again and enjoy daily life. I hope my story helps other lupus patients, especially those with stomach problems like mine. I gave my consent for my case to be reported so that more doctors learn about this option.”

## Discussion

### Clinical characteristics of LMV and limitations of conventional therapy

LMV is driven by immune complex deposition and complement activation within the mesenteric microvasculature, leading to bowel wall edema and potentially fatal ischemia if untreated (3). CT remains the diagnostic modality of choice, with hallmark findings including segmental bowel wall thickening (target sign) and mesenteric vascular engorgement (comb sign) (3, 15). Our patient’s diagnosis of lupus enteritis was based on typical clinical presentation (acute abdominal pain, vomiting, diarrhea) and characteristic CT findings (segmental ileal wall thickening with fat stranding and mesenteric vascular engorgement) in the setting of active SLE with hypocomplementemia and hemolysis. Histological confirmation was not obtained, and the initial CT was non-contrast; however, the rapid clinical and radiological response to immunosuppressive therapy further supports the diagnosis, which remains clinically defined.

Standard therapy for LMV comprises high-dose intravenous GCs, often combined with CYC or MMF, plus supportive measures including bowel rest and empiric antibiotics (3, 15). Although prompt immunosuppression frequently achieves remission, sustained GCs dependence remains a pervasive clinical problem. Cumulative GCs exposure in SLE has been independently associated with cataract, osteoporosis, avascular necrosis, cardiovascular disease, and diabetes mellitus (4), creating a therapeutic paradox in which successful induction of remission does not guarantee safe long-term maintenance.

### The rationale for B-Cell targeted therapy in SLE and LMV

B lymphocytes and their regulatory cytokines, BLyS and APRIL, play a central pathogenic role in SLE by driving autoantibody production and perpetuating immune dysregulation (5, 6). Telitacicept, a recombinant TACI-Fc fusion protein, simultaneously neutralizes both BLyS and APRIL, thereby suppressing a broader spectrum of B-lineage cells—from transitional and mature B cells to long-lived plasma cells—offering a theoretical mechanistic advantage over selective BLyS inhibition (6, 8).

### Key clinical evidence supporting telitacicept in severe SLE

The phase 2b dose-ranging trial demonstrated significantly higher SLE Responder Index 4 (SRI-4) response rates with telitacicept versus placebo (75.8% for the 240 mg dose at week 48 vs. 33.9%) (9). The subsequent pivotal phase 3 randomized controlled trial (n=335) confirmed that telitacicept 160 mg weekly achieved a significantly higher modified SRI-4 response at week 52 compared with placebo (67.1% vs. 32.7%; adjusted difference 34.5 percentage points; P<0.001), with a greater proportion of patients reducing GCs dose to ≤7.5 mg/day or by ≥25% (44.9% vs. 34.7%) and a longer median time to first flare (198 vs. 115 days) (10).

Real-world evidence has further corroborated these findings. A multicenter retrospective study of 61 SLE patients reported stepwise increases in SRI-4 response rates from 52.5% at week 4 to 80.0% at week 52, accompanied by significant complement normalization and GCs dose reduction (11). A single-center study of 38 patients similarly demonstrated significant reductions in SLE Disease Activity Index (SLEDAI) scores (from median 8.50 to 2.00) and a 41.2% rate of ≥25% GCs dose reduction (16). Most relevant to the present case, Cheng et al. specifically evaluated telitacicept in 22 SLE patients with hematological involvement, demonstrating significant hemoglobin improvement from 100 ± 19 g/L to 125 ± 22 g/L (P<0.001) after a median of 10.4 months of therapy, with only one urinary tract infection reported (12). Furthermore, Wang et al. recently reported the first case of telitacicept use specifically for LMV in a 39-year-old female with concurrent LMV and class V lupus nephritis; the addition of telitacicept 160 mg/week led to rapid abdominal pain resolution and radiological improvement within one week, enabling maintenance on prednisone 8 mg/day (14). These data provided a direct clinical rationale for the use of telitacicept in our patient.

### Interpretation of telitacicept’s role in the present case

Accurate delineation of the therapeutic timeline is essential. CTE improvement was documented on January 10, 2026—after pulse MP (500 mg/day) and IVIG (20 g/day) administered from January 4 to 8, but before telitacicept initiation on January 13. Thus, induction of remission for acute LMV and AIHA is attributable to conventional pulse therapy, which rapidly reversed hemolysis (hemoglobin rose from 61 g/L to 108 g/L).

Following the introduction of telitacicept, glucocorticoids were successfully tapered without disease flare, although concomitant TAC and HCQ may have contributed to the response. After telitacicept was added, prednisone was tapered from 60 mg/day at discharge to 10 mg/day over approximately 6 months without any recurrence of abdominal symptoms or hemolysis. This outcome is particularly striking given that multiple prior attempts to optimize non-biologic immunosuppression had been complicated by gastrointestinal intolerance to MMF or inadequate response to cyclosporine and TAC monotherapy. Hemoglobin progressively normalized to 132 g/L, serum albumin rose from 34.2 g/L to 42.4 g/L, and complement C3 and C4 normalized (0.9 g/L and 0.22 g/L, respectively). Coinciding with the scheduled tapering plan, prednisone was reduced from 50 mg/day to 40 mg/day on March 14, following a mild upper respiratory infection that had resolved with temporary interruption of telitacicept and TAC and a short course of levofloxacin.

The sustained normalization of complements and hemoglobin during rapid GCs withdrawal, together with the absence of flares, suggests that the combination regimen—including telitacicept, TAC, and HCQ—may have provided effective control of the underlying autoimmune diathesis that had previously made tapering difficult. This aligns with the 2023 EULAR recommendations advocating early biologic use to minimize GCs exposure and improve long-term outcomes (13). Persistent DAT positivity (4+) despite clinical stability and normalized hemoglobin is a well-recognized dissociation also observed with other biologics; it likely reflects residual low-level autoantibody binding insufficient to cause hemolysis once the inflammatory milieu is controlled, and should not be equated with active AIHA. From a treat-to-target perspective, although the patient achieved a substantial reduction in glucocorticoid dose (from 60 to 10 mg/day) and remained clinically stable, the current prednisone dose of 10 mg/day still exceeds the thresholds for LLDAS (≤7.5 mg/day) and DORIS remission (≤5 mg/day) as defined in the respective frameworks (17, 18). Therefore, formal LLDAS or DORIS criteria were not met. Nonetheless, the successful taper from 60 to 10 mg/day without flare during a 6-month period represents a clinically meaningful reduction in glucocorticoid exposure, which aligns with the EULAR recommendations to minimize steroid burden (13).

### Safety considerations and infection risk

Infection remains the leading cause of death in SLE (19), and adding biologic agents to background immunosuppression raises concerns about additive infectious risk (19, 20). In the phase 3 trial, adverse events considered treatment-related occurred in 74.9% of telitacicept-treated patients versus 50.0% with placebo; URI (31.7% vs. 19.0%), reduced serum IgG (15.6% vs. 1.2%), and injection-site reactions (12.6% vs. 0.6%) were more frequent with telitacicept (10). Importantly, serious adverse events were numerically lower in the telitacicept group (7.2% vs. 14.3%) (10). In our patient, only one mild URI occurred, resolving uneventfully with temporary drug interruption and antibiotics. During the 6-month observation period, no serious adverse events were observed. This observation is consistent with the theoretical expectation that dual BLyS/APRIL inhibition may modulate B-cell function without complete pan-B-cell depletion (6), but direct comparative safety conclusions cannot be drawn from a single case.

### B-cell-targeted therapies and clinical implications

The therapeutic landscape for severe SLE has expanded with belimumab and the off-label use of rituximab in refractory disease, although randomized controlled trials of rituximab in SLE (EXPLORER and LUNAR) did not meet their primary endpoints (21, 22). More recently, obinutuzumab has shown positive phase 3 results in lupus nephritis (REGENCY trial) and active non-renal SLE (ALLEGORY trial), but these data also do not provide specific evidence for LMV or AIHA (23, 24). LMV was not explicitly represented in the pivotal telitacicept trials, and published evidence for this specific manifestation remains limited to a single prior case report (14).

### Limitations

Several limitations warrant acknowledgment. First, as a single case report, causal inferences cannot be established and findings may not be generalizable. Second, concomitant TAC, MMF (added in May 2026), and HCQ may have contributed synergistically. The temporal relationship—clinical stability maintained after telitacicept introduction in a patient who had previously shown inadequate responses to other agents—suggests an association, but does not establish a dominant role for any single agent. Third, the 6-month follow-up precludes conclusions regarding long-term efficacy and durability. Fourth, anti-dsDNA negativity limits serological monitoring, and the persistently positive DCT suggests incomplete immunological quiescence whose significance remains uncertain. Fifth, histological confirmation of LMV was not obtained, though clinical and imaging criteria strongly support the diagnosis (3, 15). Finally, formal patient-reported outcome measures were not collected.

## Conclusions

This case suggests that telitacicept, when added to a background regimen of TAC, MMF, and HCQ after induction with pulse MP and IVIG, may serve as a useful steroid-sparing adjunct during the maintenance phase in severe LMV with AIHA. The dual inhibition of BLyS and APRIL offers a mechanistic rationale for its use, but controlled data specific to LMV and AIHA are lacking. To our knowledge, this is the second reported case of telitacicept for LMV and the first to document concurrent AIHA resolution. The “induction with pulse MP followed by telitacicept-containing combination maintenance” strategy described herein warrants prospective investigation.

## Data Availability

The raw data supporting the conclusions of this article will be made available by the authors, without undue reservation.
